# Medicinal plants used in traditional medicine by Oromo people, Ghimbi District, Southwest Ethiopia

**DOI:** 10.1186/1746-4269-10-40

**Published:** 2014-05-08

**Authors:** Balcha Abera

**Affiliations:** 1 Department of Biology, College of Natural Sciences, Jimma University, Jimma, Ethiopia

**Keywords:** Ethnobotany, Traditional knowledge transfer, Preference ranking, Oromia, Ethiopia

## Abstract

**Background:**

Ethiopia is one of the six centres of biodiversity in the world with several topographies, climatic conditions and various ethnic cultures. Ethnobotanical study is a real and encourageable in rich biological resource areas for medicinal plant identification, documentation, ranking, conservation and sustainable usages. The purpose of this study was to identify the most effective medicinal plants for specific treatment through priority ranking and to assess the status of the transfer of Traditional Botanical Knowledge (TBK) based on age groups and educational levels.

**Methodology:**

Ethnobotanical data were collected using field observation and semi-structured interview, A total of 30 key informants and 165 community members were interviewed and data on medicinal plant species and associated knowledge were recorded, quantified and verified using several preference ranking methods.

**Results:**

The study revealed a total of 49 medicinal plant species (belonging to 31 families and 46 genera) used to treat various human ailments, the majority of which 40 (81.6%) species were collected from wild while the rests from home garden. Herbs constituted the largest growth habit (18 species, 37%) followed by trees (16 species, 32%) and shrubs (15 species, 31%). Leaf `17 (35%) is the plant part widely used followed by root 13 (27%), leafy-stem 5 (10%), and seed 6 (12%). Oral administration was the dominant route (63%), followed by dermal route (22%) and nasal (11%). The highest number of plant species being used for infectious (48%) followed by two or more diseases and non-infectious disease. Of five and seven medicinal plants of preference ranking the highest ranks were given first for *Croton macrostaychus* used for malaria treatment and for *Prunus africana* as ‘’rare” for immediate collection and use in the traditional treatment. Significantly higher average number of medicinal plants (p < 0.05) were reported by informants of higher institution (14.3 ± 34) and adult age groups (11.6 ± 43).

**Conclusion:**

The Ghimbi people possess rich ethno-medicinal knowledge. This study can be used as a basis for developing management plans for conservation, sustainable use and drug development.

## Introduction

About 85% of world population uses herbal medicines for prevention and treatment of diseases, and the demand is increasing in developed and developing countries [1]. Some 25% of drugs contain compounds obtained from higher plants [2]. Moreover, the investigation of herbal drugs from plants to treat AIDS, cancer, and malaria, chronic complaints such as rheumatism, arthritis and asthma have been reported [3-5]. Herbal remedies are enjoying widespread popularity throughout the world [6,7]. However, only 10% of medicinal plant species is cultivated today while the larger majority being left under wild stands threat [8,9].

Ethiopia is endowed with a diverse biological resources including about 6, 500 species of higher plants, with approximately 12% endemic, hence making it one of the six plant biodiversity rich regions [10]. Of these, more than 62.5% of the forest area are found in southwest region of Ethiopia [11] where most of the medicinal plants are confined [12] and have been used as a source of traditional medicine to treat different human and livestock ailments [13,14]. Use and management of many medicinal plants in Ghimbi district has been reported by [15]. However, this former study does not provide sufficiently detailed information on the status of Traditional Botanical Knowledge (TBK) transfer from generation to generation based on Oromo Gada system age groups and educational levels as wells as on the ranking of most potential medicinal plants for specific disease treatment in the study area. The present study was therefore to identify those potential and popular medicinal plant species used for the treatment of various diseases in Ghimbi area by Oromo community.

### Description of the study area

Ghimbi District is situated in West Wollega Zone, Oromia National Regional State, 441 km southwest of Addis Ababa, bounded by East Wollega zone in the East, Lalo Assabi district in West and Guyi district in South and Amhara Reguional estate in the North. Astronomically, the district is located between 90^0^10_9^0^17^1^ North latitude and 35^0^ 44^1^ _36^0^ 09^1^ East longitude. Generally the district has a total area of 1172 Km^2^.

Ghimbi is one of the 21 districts in the Zone having 31 administrative peasant associations or Kebels. Ghimbi is the Capital of the District. Metrological data taken from National Metrology Service (Addis Ababa) indicates that the major rainy seasons in the district include spring (May), summer (June-August) and autumn (September –October). Average annual temperature of the district is about 20°C while average annual rainfall of the district was 600-12000 mm. The study area is found within the range of 1600- 2500masl. This variation in altitude resulted in variability in climate, vegetation types, and cropping system.

Based on the 1994 National census the total population of the District was about 79, 313 of which 38, 976 are males and 40, 337 are females. Rural peoples of the District lead their life on cropping and livestock rearing. The major ethnic composition of population of the District is Oromo (99%), Amhara, and Guraghe, and other ethnic groups of very few populations. Afan Oromo which is the official language of the state region is the most (major) spoken language in the zone. Amarigna (the second most spoken language in the zone), and Guragigna are also spoken by few populations in the zone [16]. According to Ghimbi District Health Station the healthcare coverage of the study area was 70% and the major disease categories recorded by the office (2007–2008) were infectious, metabolic, nervous and spiritual specifically such as malaria, stomachace, Gastritis, Diarrhoea, sexually transmitted diseases, cancer, Tuberculosis (TB), skin infection, blood pressure, Anaemia and other helmenthsis and conjunctivitis.

## Materials and methods

### Reconnaissance survey and study site selection

**Figure 1:**
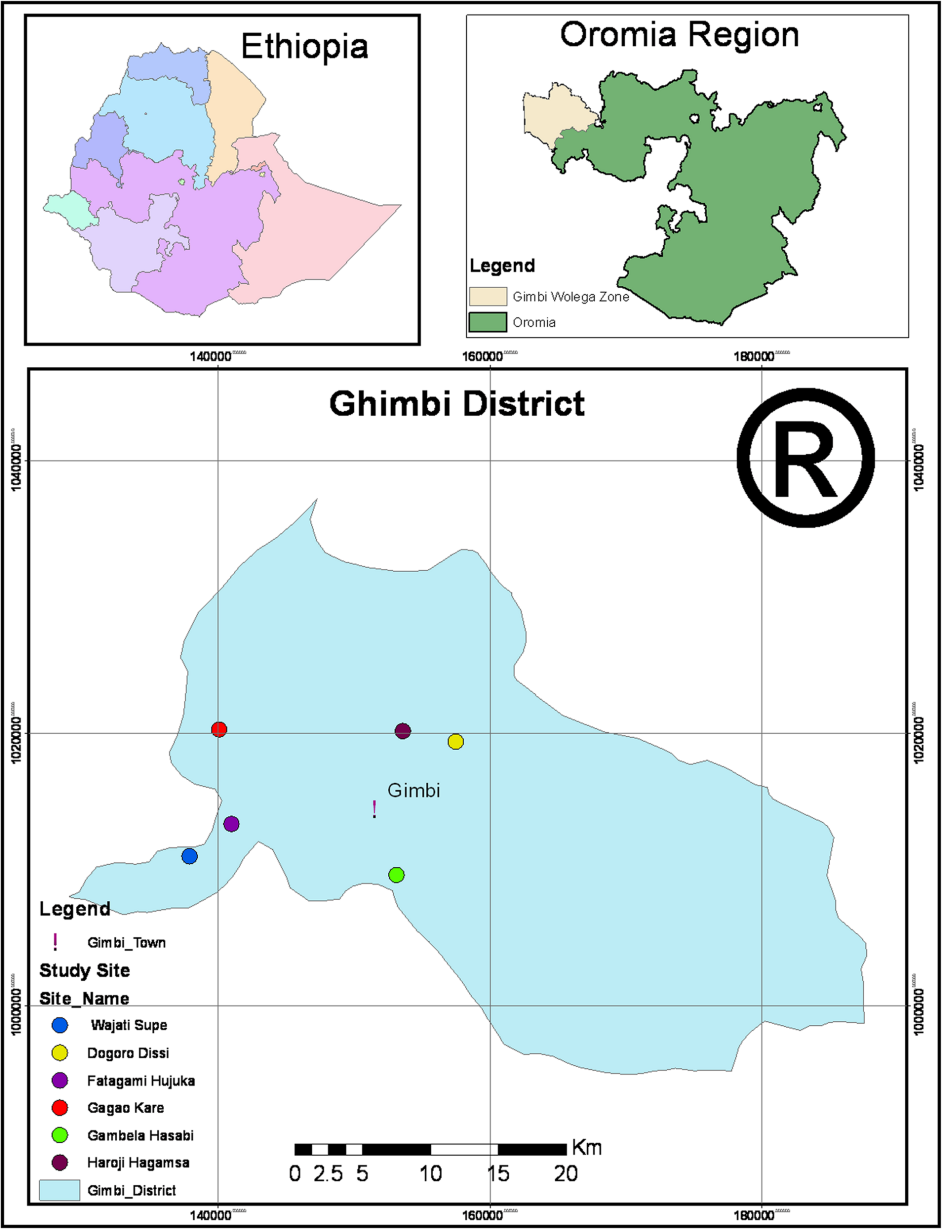
Map of the study area.

Prior to reconnaissance survey an official letter was received from Jimma University Ethical Review Committee (ERC) while verbal informed consent was obtained from each informant who was participating during the study period. A reconnaissance survey was conducted from September 1–30, 2009 in Ghimbi District (Figure 1) and determined to include six study sites (peasant associations) namely Gambella Assabi, Gaga’o Kare, Fatagami Hujuka, Wajeti supe, Dongoro Dissi, and Harojjii Hagamsa. The study sites were selected based on the prior information gathered from community leaders, knowledgeable elders, health workers, and number of traditional healers in the area, Therefore, the study was carried out in three attitudinally varying study sites. Areas with lower altitude [(1600–1800 masl) (Gambella Assabi)] is located to the southeast of Ghimbi town, medium altitude [(1800-2000masl) (Gaga’o Kare, Fatagami Hujuka, Wajeti supe)] is located to southwest of Ghimbi town, high altitude [(>2000 masl) Dongoro Dissi, Harojjii Hagamsa )] is located at the North of Ghimbi town.

### Informant selection

The informants were identified with the help of Kebele leaders, Developmental Agents (DAs) and knowledgeable elders. Thirty traditional healers (22 men and 8 women) 5 to 6 from each study site with the age of 30 and above were included as key informants to obtain pertinient information while less than 30 age groups were considered to determine the status of knowledge transfer from elders.

### Data collection

Ethnobotanical data was collected from November 2009 to February 2010 on three field trips made to the site based on methods given by [17,18]. Accordingly, semi-structured interviews and field observation with informants were employed to obtain indigenous knowledge of the local community. Interviews and discussions were based on, around a checklist of questions prepared before hand in English, and translated to Afan Oromo. Data on medicinal plants and their local names, part(s) used, methods of preparation and preservation, route of application, diseases treated and threat status were recorded at the spot with data recorder. Other relevant information were recorded by repeated inquiries at different times with the same informants to check the accuracy of information obtained and information was recorded. Finally, the specimen were collected, pressed, and dried for further identification and description.

To determine the transfer of Indigenous knowledge from generation to generation on medicinal plants use in the study area a total of 60 and 45 informants (fifteen each from 3 gada age groups (9–17, 18–24, 25–31) and from 3 educational levels (1–4, 4–8, > 8 grades) were selected from the family of traditional healers al least through naming of medicinal plants.

### Specimen identification

Preliminary identification was done in the field by using manuals and unidentified specimens were identified using herbarium materials, experts, and taxonomic keys in the various volumes of the Flora of Ethiopia and Eritrea (19–25). The collected specimens with voucher numbers, family, species and vernacular names, dates and sites of collection were recorded and deposited at the Jimma Herbarium and National Herbarium (Addis Ababa University).

### Data analysis

Microsoft Excel spreadsheet software was used to determine the proportions on growth habit, plant part(s) used, condition of medicinal plants, mode of administration and categories of diseases.

The collected ethnobotanical data on indigenous knowledge transfer (or number of medicinal plants reported by young age and educational groups) were entered into SPSS version 16.0 and summarized using descriptive statistical methods (percentage, table, chart, graphs). The presence and absence of significant differences between age and educational levels (at 95% confidence level) were checked using one way analysis of variance (ANOVA) with the assistance of software SPSS. The association between knowledge transfer versus age and educational levels were checked using Pearson chi-square test at 5% level of significance.

### Preference ranking

Preference ranking was used to rank the methods of preparation and preservation, popular medicinal plants and medicinal plants used in the treatment of malaria in the study area [17]. Each variable was ranked by 12 selected informants. All informants were oriented on each variable and asked to mark the highest value (10) for most preferred and the lowest value (1) for the least preferred on preparation and preservation methods, degree of popular medicinal plants and the healing potential of individual medicinal plants (used in the treatment of malaria). Finally, the values were summed up; ranked and illustrated using tables.

### Paired comparison

Pair comparison was used for evaluating the degree of preference of 6 selected medicinal plants reported from the study area in treating Gonorrhoea (Table 1). A list of pairs of selected items with all possible combinations was made and sequence of the pairs and the order within each pair was randomized and presented to selected informants following Martin (17) and their responses were recorded and total scores was summed using the following formula: .n (n-1)/2, n = the number of medicinal plants being compared.

**Table 1:** Medicinal plant used for the treatment of human diseases; scientific name, local name, Habit, part(s) used, method of preparation, administration route and diseases treated

***Voucher number***	**Family, Genera, species names**	**Local names**	**Growth habit**	**Plant type, cultivated/wild**	**Part(s) used and preparation method**	**Administration route**	**Disease(s) treated**
BA	Alliaceae, *Allium sativum* L.	Qullubbii	H	D	**Bulb** of *A. sativum* and rhizome of *Ginger officinale* are pounded and eaten with honey.	Oral	Malaria
*78*							
BA	Aloaceae*, Aloe monticola* Reynolds	Hargisa	H	W	**Root** is pounded and mixed with cold water and local alcohol (tella)	Oral	Anthrax
*45*							
**BA**	*Aloaceae*, *Aloe macrocarpa* Tod	Hargisa	H	W	Leaf of *A. macrocarpa* is powdered and mixed with honey	Oral	Wart
**52**							
BA	Amaranthaceae, *Amaranthus caudatus* L.	Iyyaasuu	H	W	**Leaf** is pounded and boiled.	oral	Diarrhea
*6*							
BA	Apocynaceae, *Carissa spinarum* L.	Hagamsa	SH	W	**Fresh root** of *C. spinarum is* pounded and mixed with tella (local alcohol).	Oral	Impotence
*24*							
							Gonorrhea,
							Stomachache
							Headache
BA	Asteraceae, *Acmella caulirhiza* Del.	Gutichaa	H	W	**Flower***of A. Caulirhiza* is chewed and spitted on tonsillitis	Oral	Tonsillitis
*21*							
BA	Asteraceae, *Artemisia abyssinica*	Ariti	H	W	Crushed **fresh root** of *A. abyssinica* is homogenized in water and the patient smell and drink	Oral Nasal	Evil spirit
*35*							
	Sch.Bip.ex.Rich/						
***Voucher number***	**Family, Genera, species names**	**Local names**	**Growth habit**	**Plant type, cultivated/wild**	**Part(s) used and preparation method**	**Administration route**	**Disease(s) treated**
BA	Asteraceae, *Echinops kebericho*, Mesfin	Qabarichoo	H	W	Pounded dry **root** is mixed with coffee	Oral	Toothache
*77*							
							vomiting
							Headache
BA	Asteraceae, *Guizotia scabra* (Vis.) Chiov.	Adaa	SH	W	**Leaf** of *G. scabra* is squeezed and its drop is prepared.	Dermal	Wound
*53*							
BA	Brassicaceae*, Coronopus didymus* (L.) Sm	Surumaa	SH	W	**leafy-stem***of C. didymus* is collected and dried in sunlight, crushed and mixed with soup of sorghum	Oral	Bone fracture
25							
BA	Cactaceae, *Opuntia ficus -indica* (L.) Mill.	Nimi	T	W	leaf of *O. Ficus-indica* is collected with small node and fumigated in the house	fumigation	Kill malarial vectors
*38*							
					
***Voucher number***	**Family, Genera, species names**	**Local names**	**Growth habit**	**Plant type, cultivated/wild**	**Part(s) used and preparation method**	**Administration route**	**Disease(s) treated**
BA	Capparidaceae, *Crateva adansonii* D.C. Prodr	Qolladii	SH	W	**Dried root** of *C. adansonii* powdered, mixed with water	Oral	Gonorrhoea
*4*							
BA	Capparidaceae, *Ritchiea albersii* Gilg	Arbuu	T	W	Drops from **stem** and tied on the wound **Seed** pounded and mixed with tea/ H_2_O	Oral	Cough
*27*							
BA	Combretaceae, *Combertum paniculatum* Vent	Baggii	SH	W	**Bark** latex of *C. paniculatum* is pounded and mixed with soda and creamed on affected skin.	Dermal	Ringworm
*7*							
***Voucher number***	**Family, Genera, species names**	**Local names**	**Growth habit**	**Plant type, cultivated/wild**	**Part(s) used and preparation method**	**Administration route**	**Disease(s) treated**
BA	Crassulaceae, *Kalanchoe densiflora* Rolfe	Endahula	H	W	Leaves of *K.densiflora* is squeezed, and its drop is dropped on the wound	Dermal	Gonorrhea
*61*							
BA	Cucurbitaceae, *Cucurbita pepo* L.	Buqqee	H	D	**Seed** powder is mixed with water and filtered	Oral	Gonorrhea
*67*							
BA	Euphorbiaceae, *Croton macrostachyus* Del.	Bakkannisa	T	W	Powdered **leafy-stem** of *C. macrostachyus* is mixed with H_2_O and butter and filtered finally.	Dermal	Wound
*38*							
						Oral	Malaria
						Oral	Gonorrhea
BA	Euphorbaceae, *Euphorbia abbyssinica* J.F.Gmel.	Adaamii	T	W	**Bark** decoction is taken	Oral	gastro-intestinal, Ascaris, Gonohhorea
36							
BA	Euphorbiaceae, *Justicia schimperiana* (Nees) T. Anderson	Loomii	T	D	**Seed** of *J. Schimperiana* is crushed and mixed with water and filtered	Oral	Rabies
*42*							
BA	Fabaceae, *Acacia abyssinica* Hochst ex.Benth.	Laaftoo	T	W	**Leaf** of *A. abyssinica* is smashed and the sap is made.	Dermal	Goiter
26							
BA	Fabaceae, *Albizia schimperiana* Oliv*.*	Imalaa	T	W	**Root** of *A. schimperiana and Pterolobium stellatum* is dried and powdered.	Nasal	Evil eye
*65*							
					**Root** of *A.schimperiana* is powdered and the powder is rolled in clean cloth and tied to the neck of equines.		Swelling
***Voucher number***	**Family, Genera, species names**	**Local names**	**Growth habit**	**Plant type, cultivated/wild**	**Part(s) used and preparation method**	**Administration route**	**Disease(s) treated**
BA	Fabaceae, *Calpurnia subdecandra* (L’Herit.) Schweick.	Ceeqaa	SH	W	**Leaf** of *C. subdecandra* is smashed and rubbed on affected area*.*	Dermal	Skin diseases
*28*							
BA	Fabaceae, *Erythrina abyssinica* Lam. Ex. DC.	Beroo	T	W	Crushed fresh **bark** is homogenized in water	Oral	Abdominal distention, and cramp
*63*							
BA	Fabaceae, *Taverniera abyssinica* A. Rich*,*	Dingatanya	SH	W	Dried **root** is fumigated only dry root is tuting with teelth	Oral	Spiritual disease
*18*							
							Internal Parasite
BA	Lamiaceae, *Ajuga integrifolia*, Buch.-Hamn.	Armaguusa	H	W	**Leaf** of *A. integrifolia* is pounded and mixed with nut oil	Oral	Epilepsy
*29*							
BA	Lamiaceae, *Clerdendrum myricoides* Hochst	Maraasisa	SH	W	Leaves of *C. Myricoides* are extracted with cold water	Oral	Abdominal distension
32							
BA	Lamiaceae, *Ocimum gratissimum* L.	Damakase	SH	w	**Leaf***of O. gratissimum* is squeezed and its drop is prepared	Oral	Alergic
*11*						Nasal	
						Skin	
BA	Lamiaceae, *Ocimium lamifolium* Hochst. Ex. Benth.	Hancabbii	H	D	Leaf of *Ocimum lamifolium* is smashed and sniffed	Nasal	Headache
*15*							
***Voucher number***	**Family, Genera, species names**	**Local names**	**Growth habit**	**Plant type, cultivated/wild**	**Part(s) used and preparation method**	**Administration route**	**Disease(s) treated**
BA	Loganiaceae, *Buddleja polystachya* Fresen.	Hanfaaree	T	W	**Leaf** of *B. polystachya* is chewed and spitted on cattle eye.	Optical	Eye disease
*31*							
BA	Melianthaceae, *Bersama abyssinica* Fresen.	Lolchiisaa	SH	W	**Leafy-Stem** tip of *B. abyssinica* is squeezed and creamed on wound	Dermal	Wound
*12*							
BA	Moraceae, *Ficus sycomorus* L.	Odaa	T	W	Sap is collected from **bark** surface *of Ficus sycomorus* and creamed on skin.	Dermal	Hepatitis
*47*							
BA	Muluginaceae, *Glinus lotoides* L.	Mataharree	H	W	**Leafy-stem of***G. lotoides* is crused, pwodered and liquified.	Oral	Tape worm
*23*							
							
BA	Myrsinaceae, *Maisa lanceolata*,	Abbayii	T	W	**Bark** of *M. lanceolata* is pounded and mixed with butter	External,	Elephantiasis
*82*							
BA	Myrtaceae, *Eucalyptus globules* labing	Bargamoo Adii	**T**	**D**	**Leaf** of *E. globules* is boiled in water	Nasal	Influenza
*48*							Allergic
BA	Poaceae, *Cynodon dactylon* L. Pers	Coqosa adii	H	W	**Leafy-stem** is harvested and given for cattle	Oral	Bone fracture
*30*							
BA	Poaceae, *Cynodon nemfuensis*	Coqorsa gurraacha	H	W	Crash **leaf stem** with teeth	Dermal	Tonsillitis
*46*							
							
BA	Phytolaccaceae*, Phytolocca dodecandra*, L ‘Hert.	Andoodee	H	W	**Leaf** of *P. dodecandra* is squeezed and juice is made	Oral	Sinus
*34*							
							Anemia
BA	Plumbaginaceae, *Plumbago zeylanica* L.	Martus	H	W	**Leaf** of *P. zeylanica* is squeezed and juice is made	Oral	Cancer
*41*							
BA	Polygalaceae, *Rumex neppalensis* Spreng	Tult	H	W	**Root** of *R. nepalensis* is pounded and two cup of tea is taken with coffee.	Oral	Stomachache
*10*							
BA	Polygalaceae, *Securidica longipedunculata* Fresen	Etsamanaay (Amharic)	T	W	**Root** is pounded and mixed with H_2_O	Oral	Intestinal parasite
*13*							
BA	Ranunculaceae, *Nigella sativa* L.	Gurra	SH	D	Concoction, dry pounded **seed** with pounded dry *Brassica juncea and Echinops kebericho* root, powder is mixed with water	Nasal	Headache
*22*							
BA	Rocaceae, *Prunus africana* (Hook. f.) Kalkman	Hoomii	T	W	Liquid extracts from *P. africana* bark is pounded, juiced and drunk for treatment	Oral	benign prostatic hyperplasia, prostate gland hypertrophy
*37*							
BA	Rubiaceae, *Coffee arabica* L.	Buna	T	D	Roust the **seed**, pounded and mixed with honey	Oral	Diarrhea
*20*							
***Voucher number***	**Family, Genera, species names**	**Local names**	**Growth habit**	**Plant type, cultivated/wild**	**Part(s) used and preparation method**	**Administration route**	**Disease(s) treated**
BA	Rutaceae, *Clausena anisata* (Wild.) Benth.	Ulumaa’i	T	W	**Leaf** of *C. anisata*, *Solanecio gigas* and *Justicia schimperiana* are pounded together	Dermal	Skin irritation
39							
BA	Rutaceae, *Ruta chalepensis* L.	Cilaattama	H	D	**Leaf** of *R. chalepensis* and leaf of *Vernonia amygdalina* are smashed together and one cup of domestic alcohol is taken by human	Oral	Stomacache
*44*							
BA	Simarobouceae, *Brucea antidysentrica* Fresen	Qomonyo	SH	W	**Leaf** of *B. antidysenterica is* pounded and mixed with water in dish.	Dermal	External parasite
44							
BA	Solanaceae, *Datura stramonium* L.	Asaangira	SH	W	**Leafy-stem** is squeezed and its drop prepared with butter	Dermal	Wart Toothache
*56*							
BA	Solanaceae*, Withania somnifera* L. Dunal	Kumo	SH	W	**Leaf of***W. somnifera is* powdered, juiced and drunk for 4 days.	Oral	Malaria
55							
BA	Zingibiraceae, *Zingiber officinale* Roscoe	Zinjibila	H	**D**	**Leafy-Stem** is pounded and mixed with “kullubbii + gurra”	Nasal	Influenza Internal Parasite treatment
*17*						Oral	

**Keys:** H- Herb, SH-Shrub, T-Tee, W, Wild. D-Domesticated.

BA= Balcha Abera

### Fidelity level index

Fidelity level index (FL) is used to quantify the importance of a given species for a particular purpose in a given cultural group. In this study, FL was used to determine the relative healing potential of 4 medicinal plants against human ailments based on the proportion of informants agreement on the use of a given medicinal plant against a given ailment category. The formula used was as follows: FL% = lp/lu × 100, FL% - Percentage of Fidelity Level, lp – the number of informants who independently indicated the use of the species for the same major ailments, lu – the total number of informants who mentioned the plant for any major ailment [19].

## Results

### Medicinal plant species diversity and origin

A total of 49 medicinal plants were reported by the local healers from the study area as being used for treatment in the area. These plants are distributed in 43 genera and 31 families. Family Fabaceae was represented by 5 species followed by 4 species of Asteraceae and Lamiaceae each and 3 species of Euphorbiaceae. Six families were represented by 2 species while the remaining families were represented by one species. Of these, 40 (81.6%) species were reported from the wild while the rests were from home garden cultivated by the community (Table 1).

### Growth habit

Analysis of growth forms of these medicinal plants revealed that herbs constitute the largest category (18 species, 37%) followed by trees (16 species, 32%) and shrubs (15 species, 31%).

### Plant part(s) used

Plant part(s) used for medicinal purposes indicated that Leaf 17 (35%) is the plant part widely used followed by root 13 (27%), leafy-stem 5 (10%) and seed 6 (12%), while the rest include bark 5 (10%), flower 1 (2%), bulb 1 (2%), and tuber 1 (2%).

### Medicinal plants condition

The local healers of the study area employ several collections of plant conditions. Thirty four (44.8%) preparations are made from fresh form, followed by dry 27 (35.5%) and both dry and fresh 15 (19.7%).

### Knowledge of local healers on preparation methods

The local healers employed several methods of preparation of traditional medicines from plants. Powdering and pounding were the most frequently used methods of traditional medicine preparation in the study area as ranked by healers (Table 2). According to the local healers, both pounding and powdering as a strategy permit to preserve the plant materials that are not available both in dry and rainy seasons. It was also cited that, these are effective for the complete extraction of the potential content of the plant and increase the curative power of the medicine or its efficacy, as both increases the healing power of the remedy through faster physiological reaction. After preparation, the remedies are either used soon or preserved for latter use.

**Table 2:** Traditional medicinal plant preparation methods ranked by 12 local healers in the area

**Preparation methods**	*LH1	**LH2**	**LH3**	**LH4**	**LH5**	**LH6**	**LH7**	**LH8**	**LH9**	**LH10**	**LH11**	**LH12**	**Total**	**Rank**
Powdering	10	9	8	7	9	9	10	10	8	9	10	9	108	1^st^
Pounding	7	6	8	7	7	8	8	7	7	8	6	8	87	2^nd^
smashing	5	5	6	7	5	5	6	4	6	5	7	7	68	3^rd^
Squeezing	4	-	6	6	4	3	5	3	4	5	4	6	50	4^th^
Chewing	3	4	2	5	3	2	2	3	3	4	5	6	42	5^th^
Crushing	3	2	1	3	2	2	1	2	3	3	2	4	37	6^th^
Dry bath	2	1	2	2	1	1	3	1	2	1	3	3	22	7^th^
Stem bath	1	3	-	-	-	-	1	1	1	-	-	2	9	8^th^

*LH- Local Healer.

### Traditional medicinal plants preservation methods

The use of plastic bags was ranked 1st by traditional practitioners for the preservation of medicinal plants followed by clay-made containers, cloths sheet, roof hanging and sealed bottles (Table 3).

**Table 3:** Preference ranking on the knowledge of 12 local healers on preservation methods of medicinal plants

**Preservation methods**	*R1	**R2**	**R3**	**R4**	**R5**	**R6**	**R7**	**R8**	**R9**	**R10**	**R11**	**R12**	**Total**	**Rank**
Clay-	5	5	4	4	3	5	5	4	4	5	5	5	54	2
Container	5	5	5	5	4	4	5	5	5	5	5	5	58	1
Plastic bags	3	4	5	3	4	3	3	3	2	4	4	4	42	4
Roof hanging	4	4	4	3	5	4	5	3	5	3	5	5	50	3
Cloths sheet	2	1	1	2	2	1	2	1	2	2	1	3	20	5
Sealed bottles														

*R = Respondent.

According to the discussions made with traditional healers the preparations made drawn from mixtures of different plant species with different additive substances like honey, sugar, teff flour, butter, soda ash, salt, ground honey, soil and charcoal ash for the treatment of single ailment (data not given). These additive substances had been reported to have double function i.e. to improve flavor and reduce adverse effects such as vomiting and diahrrhoea, and enhance the efficacy and healing conditions.

### Indigenous knowledge on the mode of administration

Methods of administration of traditional medicinal plants prepared products by the local healers/community. The major routes of administration in the study area were reported to be oral, dermal, nasal, anal, auricular and optical. Oral administration was the most cited route (63.9%), followed by dermal route (23%) and nasal (10%). Both oral and nasal routes (4%) permit rapid physiological reaction of the prepared medicines with the pathogens and increase its curative power.

### Medicinal plants popularity in the community

The degree of agreement on ranking of ten medicinal plants based on the principle that the plant species are widely known and frequently used for the treatment of a particular ailment among the local community is indicated in Table 4. *Glinus lotoides* scored the highest point and ranked first followed by *Echinops kebericho and Lepidium sativum* species as widely known by the large local community and even prepared and used at family level.

**Table 4:** Ranking of 10 medicinal plants known in the study area by the local community as responded by 12 informants

**Species scientific name(s)**	*R1	**R2**	**R3**	**R4**	**R5**	**R6**	**R7**	**R8**	**R9**	**R10**	**R11**	**R12**	**Total**	**Rank**
*Glinus lotoides*	10	9	8	8	9	9	10	9	9	10	8	9	108	**1**
*Echinops kebericho*	9	8	10	8	7	7	9	7	9	8	6	8	96	**2**
*Brucea antidysentrica*	5	6	7	5	8	6	6	7	6	7	6	6	75	**5**
*Embelia schimperi*	4	5	6	7	5	4	3	5	4	3	5	6	57	**9**
*Allium sativum*	8	7	6	8	6	8	10	6	8	7	8	7	89	**3**
*Taverniera abyssinica*	5	6	4	7	6	6	7	5	5	7	6	5	69	**6**
*Vernonia amygdalina*	4	5	5	6	7	4	5	6	7	4	4	5	62	**8**
*Croton macroststchyus*	6	7	5	5	6	4	6	5	5	5	5	6	65	**7**
*Ocimum gratissimum*	4	6	7	7	4	8	3	2	1	3	5	7	56	**10**
*Ocimum sanctum*	6	7	7	5	8	7	6	6	5	7	4	8	76	**4**

*R = Respondent.

### Ranking of medicinal plants for malaria treatment

The highest rank was given for *Croton macrostaychs* followed by *Alium sativum* and *Carica papaya* for malaria treatment by the local healers in the study area (Table 5).

**Table 5:** Preference ranking of 5 selected medicinal plants on their degree of treating malaria as perceived by 12 local healers

**Respondents**	***Lepidium sativum***	***Croton macrostaychs***	***Allium sativum***	***Carica papaya***	***Vernonia amygdalina***
*R1	5	5	8	5	0
R2	2	4	6	7	3
R3	0	6	4	5	5
R4	8	5	5	7	2
R5	5	9	3	6	3
R6	4	5	6	4	6
R7	2	6	5	3	5
R8	6	5	5	5	5
R9	5	7	7	6	7
R10	2	8	8	4	8
R11	3	8	6	4	6
R12	4	9	5	6	5
Total	46	77	68	62	51
Rank	5^th^	1^st^	2^nd^	3^rd^	^4th^

*R = Respondent.

### Paired comparison

Of the medicinal plants reported in the study area to treat Gonorrhea a paired compaison (Table 6) indicates that the highest rank was given for *Carissa spinarum* followed by *Cucurbita pepo* to treat Gonorrhea.

**Table 6:** Paired Comparison of 6 medicinal plants in the treatment of Gonorrhea as indicated by 12 respondents

**Species**	***R1-R12***							
*Carissa spinarum*	**-**	**Cs**	**Cs**	**Cs**	**Cs**	**Cs**	**5x**	**1**
*Crateva adonsani*	**-**	**-**	**Ca**	**Ca**	**Ca**	**Kd**	**3x**	**3**
*Croton macrostachys*	**-**	**-**	**-**	**Cp**	**Cp**	**Kd**	**0x**	**6**
*Cucurbita pepo*	**-**	**-**	**-**	**-**	**Cp**	**Cp**	**4x**	**2**
*Euphorbia abyssinica*	**-**	**-**	**-**	**-**	**-**	**Ea**	**1x**	**5**
*Kalanchoe densiflora*	**-**	**-**	**-**	**-**	**-**	**-**	**2x**	**4**

### Fidelty level index

Fidelity level (FL), as an estimation healing potential, was determined for all reported medicinal plants. Accordingly*, Lepidium sativum* and *Plumbbago zeylanica* were the plants having the highest level values, for their use to treat infectious and metaboilc diseases, each scoring 100%, followed by *Euphorbia abyssinica* (93%) (Table 7).

**Table 7:** Fidelity level values of medicinal plants cited by 12 or moe infomants for being used against a given major ailment category

**Medicinal plant**	**Major ailments category**	**Ip**	**Iu**	**FL**	**FL%**
*Lepidium sativum*	Infectious (malaria, Influenza)	12	12	1	100
*Euphorbia abbyssinica*	Sexual diseases (Gonorrhea, Syphills)	14	15	.93	93
*Croton macrostachyus*	Abdominal (Ameboic dysenty, Ascaisis, Tape worm)	13	16	.81.	81
*Plumbago zeylanica*	Metabolic (Breast cancer, blood pressure)	12	12	.1	100

### Treated disease categories versus medicinal plants

**Figure 2:**
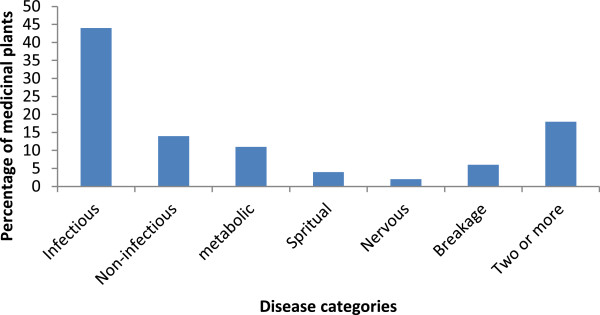
Frequency distribution of medicinal plants used in the treatment of diseases in the study area.

Of the total number of medicinal plants reported by local healers 48% (n = 24 species) were used for the treatment of infectious diseases followed by the 20% (10 species) for two or more diseases and non-infections (Figure 2).

### Medicinal plants versus degree of accessibility in the study area

**Figure 3:**
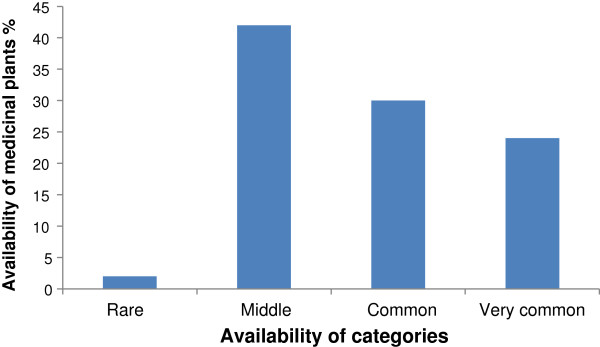
Availability of medicinal plants in the study area.

The availability status of reported medicinal plant species in the study area was analyzed using an Availability Index (AI) categories developed by [20]. Accordingly, one species namely *Prunus africana* was reported as ‘’Rare” 1 (2%) Followed by ‘’Middle” 21 (42%), ‘’common” 15 (30%), and ‘’vey common” 12 (24%) species (Figure 3).

### Knowledge transfer status among different age groups and educational levels

A significant difference (p < 0.05) was observed in naming medicinal plants between student informants (of higher institution, 9–12 Grades who were from rural) and the rest of educational levels (both from urban and rural students). Thus, significantly higher average number of medicinal plants (p < 0.05) were reported by informants of higher institution (14.3 ± 34) and 9 to12 Grades (12.12 ± 23) who were from rural than all the rest of educational levels. Significantly higher average number of plants (p < 0.05) was also reported by informants of 25 to 32 age groups (11.6 ± 43) while there was no significant difference (P > 0.05) between 9–17 and 18–24 age groups (Tables 8 and 9).

**Table 8:** Indigenous transfer of medicinal plants knowledge among different age groups of healers’ family members

**Age group**	**Age stages local name**	**Number of medicinal plants reported/informant**															**Mean**
		**R1**	**R2**	**R3**	**R4**	**R5**	**R6**	**R7**	**R8**	**R9**	**R10**	**R11**	**R12**	**R13**	**R14**	**R15**	
9-16	Foollee	1	2	1	3	2	2	2	3	2	2	4	3	2	1	1	2 ± 34^a**^
17-24	Qondaala	3	5	4	5	6	5	4	4	3		5	4	3	2	4	4 ± 34^a^
25-32	Kuusaa	14	13	16	15	8	9	6	15	16	18	13	11	8	7	5	11.6 ± 43^b^

*) Means with standard deviations within the same column followed by different letters (a-b) are significantly different (P < 0.05).

**Table 9:** Indigenous transfer of medicinal plants knowledge among family members and relative of healers with different educational level

**Educational level**	**Residence**	**R1**	**R2**	**R3**	**R4**	**R5**	**R6**	**R7**	**R8**	**R9**	**R10**	**R11**	**R12**	**R13**	**R14**	**R15**	**Mean**
0-4	Rural	4	5	6	7	4	3	6	2	4	2	4	3	5	5	4	**4.27** ± 0.14^a*^
	Urban	3	2	2	2	4	4	3	3	5	4	4	5	4	5	3	**3.53** ± + 23^**a**^
5-8	Rural	5	3	5	6	5	4	6	4	5	4	4	5	4	4	4	**5.33** ± **13**^**a**^
	Urban	3	2	5	1	4	4	6	3	5	4	2	5	4	3	4	**3.67**^**a**^ ± 34^a^
8-12	Rural	15	14	12	10	9	10	10	12	11	13	14	12	13	11	14	**12.12** ± 23^**b**^
	Urban	5	4	3	7	5	3	7	6	5	3	4	7	8	2	4	**4.9** ± 14^**a**^
Higher Institution	Rural	16	18	13	12	11	14	15	14	13	13	18	19	12	15	12	**14.3** ± 34^b^
	Urban	5	6	4	7	6	9	5	6	5	5	7	6	5	10	8	**6.31** ± 23^a^

*Means with standard deviations within the same column followed by different letters (a-b) are significantly different (P < 0.05.

## Discussion

This study revealed 49 medicinal plant species representing 31 families and 43 genera used to treat various human ailments. Of these, 13 species were in common with [21], 14 with [13], 15 with [22]. Three species that are common with [23] are known to be used in the medicinal flora of other African countries. In this study, Family Fabaceae was represented by 5 species followed by 4 species of Asteraceae, and Lamiaceae each and 3 species of Euphorbiaceae. Fabaceae, Asteraceae, and Lamiaceae are among the most represented families in the flora of Ethiopia and Eretria ( [24-26]. This study shows that the highest percentage of medicinal plants was obtained from wild while the rest were from home garden. In agreement with this study, similar percentage of medicinal plant species reported from different parts of the country by [23,27-29]) were from natural vegetation.

Analysis of growth habit of medicinal plants in this study indicated that there was no such difference especially in the proportion of herbs and trees. The distribution of similar proportion of growth forms is an indication of the harmonious growth of indigenous forest species different from the arid and semiarid regions reported by various authors [6-33].

Analysis of data on plant parts used indicated that leaf is the major part widely employed by local healers in the preparation of remedies followed by root. Previous reports in Ethiopia [15-22,24-26,30-33]. have also shown that leaves were the most commonly used to treat various health problems Given the highest frequency of leaves used for medicinal purposes in the study area threat to the destruction of medicinal plants especially to trees and shrubs was found to be minimal, as high threat to the mother plant comes with root, bark and leafy-stem harvests. However, [34] indicated that the harvest of leaves has also a threat to the deterioration of medicinal plants since the removal of leaves limits the transformation of vegetative to reproductive development sucha as flower production and fruit/seed set, which in its turn limits the natural/wild regeneration of plants.

The dependency of local people on fresh materials in the study area including the removal of fresh barks and leaves put the plants under serious threat than the dried form, as fresh materials are harvested directly and used soon with its extra deterioration with no chance of preservation i.e. not stored for latter use. However, during this survey local healers argue that fresh materials are effective in treatment as the contents are not lost before use compared to the dried forms. The livelihood of most traditional healers relied on fresh materials that had aggravated the decline of rare medicinal plants from the study area. Traditional practitioners were collecting medicinal plants with less attention than would be preferred from viewpoint of conservation of plant resource. Sofowora [23] has reported that the uses of fresh medicinal plants are more effective than other parts.

Of the major disease categories in the study area, infectious diseases are mainly treated traditionally using the large number of medicinal plants. This may be due to the distribution of various pathogens as a result of less sanitation and control measures in developing countries. This could also demonstrate the effort of local healers in searching out more and appropriate medicinal plant species for treatment of such diseases.

According to the informants, the majoity of medicinal plant parts of the study area are prepared either in combination with other medicinal plant parts or with other additives such as boiled coffee, honey and local bevereages (tella) for different purposes (either to increase the healing potential or to improve the flavour and taste or to avoid abdominal discomfort) [27,28]. For instance, a traditional medicine applied to treat tape worm infection is prepared by the combination of several medicinal plant parts ( Example, *Hagenia abyssinica*, *Glinus lotoides*) with other additives such as local beverages and salt.

As indicated by preference ranking in this study, pounding and powdeing were the most frequently used methods of remedy preparation followed by presering in plastic bags as the most suitable preservation method in the study area. According to the informants’ response different methods of remedy preparation and prespervation depend upon an equipment used, prepared and preserved plant parts, temperature and preservation period. Regarding the popular medicinal plants in Ghimbi District as ranked by informants through preference ranking method *Glinus lotoides* was the most widely known not only by the local practioniers but also among the large local community of the study area in the treatment of tapeworm infection, followed by *Echinops kebericho and Allium sativum.* In contrast, [29] have recently reported that among 9 medicinal plants *Croton macrostachys* was the most popular in the treatment of the same (tapeworm) infection followed by *Cucumis sp.* Various authors have also reported the existence of popular medicinal plants in different regions of the country such as *Cucumis pastulatus* for Tuberculosis, *Ocimum urtitolum*, *Rumex abyssinicus*, *Solanum incanum, Vernonia amygdalina* to treat ‘’michi”, gonorrhea, toothache and urine retention, respectively [27-29]. In other study reports conducted by [35] and [36] using paired comparison, preference ranking and direct matrix *Allium sativum* was found to be the most preferred in the treatment of malaria in the northern part of the country. Of the 9 medicinal plants ranked in the study conducted by [37] in Nigeria *Azadirachta indica* was reported as a prime candidate for investigation, as it recorded the highest rank by the informants. Over 1,200 plants belonging to 160 families were reported [38] to be used traditionally for the treatment of malaria. Since then the number of species has increased substantially due to the increasing worldwide interest in anti-malarial plants.

Of six medicinal plants, the highest rank was given for *Carrisa spinarum* followed by *Curcubita pepo* in the treatment of Gonorrhoea. The study conducted by [39] on antigonorrhoeal activity of some medicinal plants showed that significant antigonnorrhoeal activity was exhibited by methanol extracts of some plants, which contain bactericidal properties that stimulate the immune system to create more resistant to infection. The differences of plant species used in the treatment of the same ailment in fact depends on the availability of plant species in a particular area and cultural knowledge of particular ethnic groups.

Fidelity level (FL), as an estimation healing potential, was determined for some medicinal plants. Accordingly*, Lepidium sativum* and *Plumbbago zeylanica* were the plants having the highest level values, for their use to treat infectious and metaboilc diseases. Trotter and Logan [40], plants scoring higher informant consensus values are thought to have better potency having biologically active ingredients in treatment as compsred to plants with less informant consensus values.

The availability status of reported medicinal plant species in the study area was analyzed using an Availability Index (AI) categories developed by [20]. Accordingly, one species was reported as ‘’Rare ‘followed by ‘’Middle” 21 (45%), ‘’common” 15 (31%), and ‘’vey common” 12 (24%) species. In this study, about 47% of medicinal plant parts including root, leafy-stem and bark were reported to be used as a source of treatments. According to [22] ) medicinal plant harvests that mainly involve roots, stems and barks have serious effect on the survival of mother plants. The endangered threat status of *Prunus africana* throughout African counties resulted from the wide exploitation of its bark both as a source of traditional medicine and development of synthetic drug for the treatment of benign prostatic hyperplasis (BHP) and prostate gland hypertrophy (PGH). The elders of the local community of the study area expressed their great fear that the previous custom of replantation of forest species is declining by current generation. Medicinal plants are directly harvested and processed when only needs arise. Moreover, in Ethiopia the use of wild or uncultivated plants is a common custom and this has been accelerating the deterioration of useful plant population in addition to agricultural expansion accompanied by wide cutting original forest species and environmental degradation. Several studies also indicated [8,15,41] that a growing investment in agriculture became the major threat for the deterioration of the population of medicinal plants in general and herbs. Study by [15] also reported that the use for firewood and construction as well as agricultural expansion were the main causes for the depletion of medicinal plants in the study area.

Regarding the current transfer of indigenous knowledge in generation in the study area, this study revealed that significantly higher average number of medicinal plants (p < 0.05) were reported by informants of rural students and 25–32 age groups. This result confirmed that the traditional knowledge (TBK) is declined from elder to younger age groups. On top of this, during specimen collection, interview and field visits elders express their interest by demonstration how to collect, process, administer, and prescribe medicinal plants and with great beliefs of the traditional medicine on its effectiveness on treating the diseases while the young generation showed low participation in all aspects. Thus, decreasing positive attitude towards use of medicinal plants in traditional medicine by young generation indicate the loss of vital indigenous knowledge. Giday [40] conducted similar study using the informants of Zay Ethnic group in Ethiopia came up with 90% of the elders above 40 years possessed with enough knowledge in medicinal plants whereas 55% of the youth informants between 18 and 40 years old were without any knowledge of medicinal plants. Study conducted by [42] in Cameon reported that most young people in urban areas were not interested in the use of traditional medicine due to influence of western culture; considering that traditional medicine is superstitious, which is mainly used by poor and uneducated people. On the other hand, most of the elders kept their knowledge secrecy to generate income and to get sustainable respect from their surrounding community. More0ver, the decline of the traditional knowledge in generation is due to the due to interference of and shifts to the use of more synthetic drugs not only in the urban but also extending to the rural areas. Various reported studies indicate that the absence of formal education in traditional knowledge in developing nations is another factor for the decline of indigenous knowledge [43,44]. Moreover, most of the African modern health professionals greatly undermine the contribution of traditional medicine in health care system while the scientists of developed nations intensively search for medicinal plants to seek a solution for the old and newly rising diseases. All these factors may result to a loss of this rich and useful knowledge which has been accumulated over many generations.

## Conclusion

This study showed the wide use of medicinal plants in Ghimbi District in meeting the primary healthcare needs of the Oromo community of the study area. A limited access to modern healthcare facilities could be considered as the main factors for the continuation of the traditional practice. The medicinal plants in the area include all growth forms with almost equal proportion which could be attributed to their abundance in humid areas as compared to arid and semi arid regions. Newly harvested plant materials are mostly used in the preparation of remedies is an indication of the availability of copious plant materials in the vicinity. The medicinal plants in the study area such as *Glinus lotoides* (against tapeworm infection), *Croton macrostachyus* (against malaria) and *Allium sativum* (against malaria and other diseases) were the ones with most preferred, popular and with highest fidelity level (FL) values, an indication of their high healing potential. However, the transfer of indigenous knowledge is declining from generation to generation as a result of oral transmission. Therefore, this study recommends the argent need to incorporate this knowledge into formal education before complete lost.

## Competing interests

The author declares that they have no competing interests.
